# Longitudinal changes in resting-state functional connectivity and gray matter volume are associated with conversion to hearing impairment in older adults

**DOI:** 10.3233/JAD-215288

**Published:** 2022

**Authors:** Megan C. Fitzhugh, Judy Pa

**Affiliations:** a Mark and Mary Stevens Neuroimaging and Informatics Institute, Keck School of Medicine, University of Southern California, Los Angeles, CA, USA; b Department of Neurology, Alzheimer’s Disease Research Center, Keck School of Medicine, University of Southern California, Los Angeles, CA, USA

**Keywords:** hearing loss, hearing impairment, Alzheimer’s disease risk factor, functional neuroimaging

## Abstract

**BACKGROUND:**

Hearing loss was recently identified as a modifiable risk factor for dementia although the potential mechanisms explaining this relationship are unknown.

**OBJECTIVE:**

The current study examined longitudinal change in resting-state fMRI functional connectivity and gray matter volume in individuals who developed a hearing impairment compared to those whose hearing remained normal.

**METHODS:**

This study included 440 participants from the UK Biobank: 163 who had normal hearing at baseline and impaired hearing at follow-up (i.e., converters, mean age=63.11±6.33, 53% female) and 277 who had normal hearing at baseline and maintained normal hearing at follow-up (i.e., non-converters, age=63.31±5.50, 50% female). Functional connectivity was computed between *a priori* selected auditory seed regions (left and right Heschl’s gyrus and cytoarchitectonic subregions Te1.0, Te1.1 and Te1.2) and select higher-order cognitive brain networks. Gray matter volume within these same regions was also obtained.

**RESULTS:**

Converters had increased connectivity from left Heschl’s gyrus to left anterior insula and from right Heschl’s gyrus to right anterior insula, and decreased connectivity between right Heschl’s gyrus and right hippocampus, compared to non-converters. Converters also had reduced gray matter volume in left hippocampus and left lateral visual cortex compared to non-converters.

**CONCLUSIONS:**

These findings suggest that conversion to a hearing impairment is associated with altered brain functional connectivity and gray matter volume in the attention, memory, and visual processing regions that were examined in this study.

## Introduction

The Lancet Commission on Dementia Prevention and Care recently described that approximately 40% of dementia cases may be prevented or delayed by targeting 12 modifiable health and lifestyle risk factors [1,2]. Hearing loss in mid-life was reported to be the largest modifiable risk factor of dementia, accounting for 8% of this risk. Several studies demonstrate that there is a significant decline in cognitive performance for every 10 dB decrease in hearing ability [3-5]. This is a highly prevalent risk factor to investigate as nearly two-thirds of adults in their 70s have a hearing loss [6]. Despite the associations between hearing loss, cognitive decline, and dementia, potential mechanisms underlying these associations are not well understood.

A growing body of literature reports that hearing loss in older adults may be associated with structural and functional alterations in the brain. Most studies reveal decreases in gray matter volume and cortical thinning with increasing hearing loss in primary auditory cortex (i.e., Heschl’s gyrus) and secondary auditory regions (for reviews, see [7,8]). However, two studies show no association between gray matter volume and hearing levels [9], and one study even reports an increase in brain volume [10] in primary or secondary auditory regions.

In addition to differences in brain structure within auditory regions, hearing loss is associated with decreased gray matter volume or thickness in several higher-order cognitive areas in the frontal lobe, including medial frontal gyrus, anterior cingulate, and right insula [11-13]. Several studies also suggest that medial temporal regions (including hippocampal, parahippocampal, and entorhinal regions) exhibit reduced volume associated with hearing loss, both cross-sectionally [13-16] and longitudinally [17-19].

Beyond the effects of hearing loss on brain structure, less is known about how hearing loss affects the transfer of information between brain regions and networks. Recent studies suggest that there is increased resting-state functional MRI (fMRI) connectivity with hearing loss between Heschl’s gyrus and the anterior cingulate [20], precuneus [21], and visual cortex [22]. However, another study found decreased functional connectivity with greater hearing loss between Heschl’s gyrus and regions in the visual cortex [23]. Furthermore, the effects that hearing loss may exert on functional connectivity have only been studied cross-sectionally and it is unknown how communication across the brain may change with the progression of hearing loss.

Given the recent identification of hearing loss as a modifiable risk factor for dementia, it is important to characterize the effects of hearing loss on brain functional connectivity and brain structure to identify potential mechanisms that may underlie its association with dementia. Using a large, longitudinal dataset of older adults, this study investigated the relationships between resting-state functional connectivity, brain volumes, and hearing loss over time by comparing individuals who developed impaired hearing to those whose hearing remained normal. Primary analyses evaluated functional connectivity using Heschl’s gyrus as the seed region, with secondary analyses exploring connectivity from subregions within Heschl’s gyrus. Based on the reviewed literature, connectivity from bilateral Heschl’s gyrus to the cingulo-opercular network, the visual network, and bilateral hippocampus were selected *a priori* as regions of interest. We hypothesized that the development of a hearing impairment over time is associated with changes in functional connectivity between primary auditory and the specified regions of interest. We further hypothesized that hearing impairment would be associated with reduced brain volumes in Heschl’s gyrus and regions within our functional networks of interest.

## Methods

### Participants

Participants were selected from the UK Biobank dataset based on available hearing ability scores, resting-state fMRI data, and demographic variables of interest (e.g., age, sex) at Instance 2 (neuroimaging baseline visit) and Instance 3 (neuroimaging follow-up visit). This approach ensured there were no missing data in any of the analyses. The total sample size was 440, with an age range of 50 – 80 years at baseline (Table 1). Socioeconomic status was assessed by the Townsend Deprivation Index [24] using participant postal codes and national census data. The population mean of the Townsend Deprivation Index is zero (SD = 3.44), with positive values reflecting lower socioeconomic status. Exclusionary criteria included a history of diabetes and cancers that could affect the brain or auditory pathway (i.e. cancers of the brain, meninges, spinal cord, cranial nerves, or ear). Participants who had a cochlear implant were excluded due to the inability to obtain an MRI. Participants who wore hearing aids were included in the study (n=13). Selection of participants was further restricted to come from only a single imaging site using a single MRI scanner to minimize inter-scanner variability (site number 11025).

As described in [25] and [26], all participants provided informed consent. All procedures were overseen by the Ethics and Guidance Council (http://www.ukbiobank.ac.uk/ethics) that has developed an Ethics and Governance Framework with the UK Biobank (given in full at http://www.ukbiobank.ac.uk/wp-content/uploads/2011/05/EGF20082.pdf). Approval was also obtained from the North West Multi-center Research Ethics Committee. This research was conducted using the UK Biobank Resource under Application Number 60021. Complete details of all UK Biobank procedures are available at www.ukbiobank.ac.uk.

### Speech Reception Threshold

The Digit Triplets Test [27,28] was used to obtain speech reception threshold (SRT) scores as a measure of hearing ability. This was the only objective measure of hearing ability collected by the UK Biobank (no pure-tone audiometry was collected) and was conducted at the same visits as the MR imaging. Prior to the start of the task, participants were instructed to remove hearing aids, if present, and adjust the volume to a comfortable level. Participants wore over-the-ear headphones. During the task, participants listened to 15 sets of three digits spoken by a single female speaker. After each set, participants entered the triplet into a keypad on a computer touch screen. The triplets were presented in white noise shaped to the average spectrum of the triplets. The background noise level remained constant while the triplet volume level varied by trial: the level decreased by 2 dB with every correct response and increased by 2 dB with every incorrect response. The outcome measure used in this study was the average signal to noise ratio (SNR; i.e., the level of the triplets compared to the level of the background noise) from last 8 sets. Scores ranged from −12 to +8 dB, where a higher value (more positive) indicated poorer hearing ability. Each ear was tested separately, resulting in an SRT score for each ear.

We computed a better ear SRT variable at both the baseline and follow-up visits by using the lowest SRT score from either the left or right ear. If the score for only one ear was available, that score was used in the better ear SRT variable. All baseline SRT scores were available, while only five participants at follow-up had SRT scores from a single ear. We used the term hearing impairment in our study because levels of hearing loss are typically defined using pure tone audiometry. Previous work from the UK Biobank determined that hearing ability can be categorized into three levels using the SRT scores obtained from this task: normal < −5.50 dB, insufficient −5.50 to −3.50 dB, and poor > −3.50 dB, with insufficient and poor levels considered a hearing impairment (see [27] for further description of these cut-off scores). Scores from the better ear SRT variable were coded to reflect this categorization at each study visit. Participants in our analyses were selected with the criteria of having normal hearing at baseline. We then created a binarized variable (i.e., SRT group) to group participants into “converters,” those with normal hearing at baseline and either insufficient or poor hearing at follow-up, and “non-converters,” those who maintained normal hearing at both baseline and follow-up visits. The groups did not differ on age, sex, and other baseline demographics of interest included in our analyses (Table 1), although more individuals in the converter group wore hearing aids compared to the non-converters. Similarly, baseline SRT scores were significantly poorer in converters compared to non-converters, however, all baseline SRT scores in the converter group fell within the “normal” category by group definition.

### MRI Acquisition

MRI acquisition protocols were fully described in a previously published study [29]. Briefly, MRI data were acquired using a single 3T Siemens Skyra scanner and a standard 32-channel Siemens RF receive head coil. The T2-weighted resting-state fMRI data were collected using an EPI pulse sequence with a multiband acceleration factor of 8. Temporal resolution was 0.735s, spatial resolution was 2.4mm isotropic, and the acquisition time was 6 minutes and 10 seconds (490 volumes). EPI pulse sequences and reconstruction code used by the UK Biobank are from the Center for Magnetic Resonance Research at the University of Minnesota and were generated, in part, for the Human Connectome Project [30]. The T1-weighted structural MRI data were acquired using a 3D MPRAGE sequence with a sagittal orientation and in-plane acceleration iPAT=2. The resolution was 1x1x1 mm, the field-of-view was 208x256x256, and the acquisition time was five minutes. Further documentation is detailed at https://biobank.ctsu.ox.ac.uk/crystal/crystal/docs/brain_mri.pdf.

### Functional MRI Processing

This study utilized minimally preprocessed T2-weighted resting-state fMRI data generated by an image-processing pipeline developed and performed on behalf of UK Biobank (for complete details, see [29]). Briefly, this pipeline was conducted in FSL [31] using the MELODIC and MCFLIRT tools and included motion correction, grand mean intensity normalization by a single multiplicative factor, high pass temporal filtering, EPI unwarping using acquired fieldmaps, gradient distortion correction, removal of structured signal artifacts using ICA-FIX, and alignment to the T1-weighted anatomical image. The relative root mean squared intensity difference from one volume to the subsequent volume (i.e., dvars; [32]) was saved during the MCFLIRT motion correction phase and was used in our study as a head motion summary variable.

We applied additional preprocessing steps in FSL which included 1) removal of first 14 volumes to account for ~10s of signal stabilization, resulting in 476 volumes analyzed; 2) alignment to MNI space by applying the T1 to MNI transforms (which were saved separately in the T1 image preprocessing pipeline performed by the UK Biobank); 3) volume outlier detection using the artifact detection tool (ART, incorporated into the CONN toolbox (see below), http://www.nitrc.org/projects/artifact_detect); 4) denoising to remove signals associated with cerebrospinal fluid and white matter, and 5) spatial smoothing using a 5mm FWHM Gaussian kernel. ART computes a composite motion measure used for volume scrubbing during analysis. For each participant, the estimated maximum voxel displacement from the combined translation and rotation displacement measures was computed. The mean global signal across the entire time series was also computed and Z-transformed. Nuisance regressors were generated for each participant to scrub volumes with framewise displacement greater than 0.9mm or with global signal mean changes greater than 5 standard deviations (or approximately 3% of subjects’ data, reflecting ART’s default intermediate settings). This procedure identified a mean (sd) of 4.4 (14.7) out of 476 total volumes for scrubbing.

### Functional Connectivity Analyses and Region of Interest Selection

Functional connectivity was computed using the functional connectivity toolbox (CONN, version 19c; [33]) between seed ROIs in left and right Heschl’s gyrus and target ROIs that constitute the cingulo-opercular network, visual network, and left and right hippocampus. Heschl’s gyrus was chosen as the auditory seed region in primary analyses. Heschl’s gyrus subregions served as seed regions in secondary, exploratory analyses. All ROIs (except for the Heschl’s gyrus subregions) were pre-defined in the CONN toolbox and were non-overlapping (see Table 2). ROIs for bilateral Heschl’s gyrus and hippocampus were derived from the FSL Harvard-Oxford Atlas and network ROIs were previously generated from an independent component analysis of 497 subjects from the Human Connectome Project. Heschl’s gyrus subregion ROIs were derived from the SPM Anatomy toolbox [34] and included, from posteriomedial to anteriolateral, Te1.1, Te1.0, and Te1.2 [35]. Within each ROI and for each participant, the blood oxygen-level dependent (BOLD) signal was averaged across all voxels. Bivariate correlations of the average BOLD signals were computed between pairs of ROIs and Fisher Z-transformed. The Fisher Z-transformed correlations between seed and target ROIs were extracted for our primary analysis.

### Structural MRI Processing

This study utilized existing imaging-derived phenotypes (IDP; i.e., outcome variables of brain structure or function) generated by an image-processing pipeline developed and run on behalf of UK Biobank ([29]; for complete processing details, see https://biobank.ctsu.ox.ac.uk/crystal/crystal/docs/brain_mri.pdf). Briefly, the processing was conducted in FSL and included gradient distortion correction, brain tissue segmentation, normalization to MNI space, and FAST gray matter segmentation into 139 ROIs of gray matter partial volume estimates (category ID 1101). ROIs at this stage are defined from a combination of parcellations from the Harvard-Oxford cortical and subcortical atlases and the Diedrichsen cerebellar atlas. Our structural MRI analyses included ROIs that were either the same anatomical ROIs as our functional connectivity analysis (e.g., Heschl’s gyrus and hippocampus) or ROIs which approximately spatially overlapped with the functional network ROIs from the connectivity analysis (i.e., insular cortex, anterior cingulate cortex, and inferior lateral occipital cortex). We also used the provided IDP (field ID 25010) for total brain volume, calculated as the sum of the gray and white matter volumes.

### Statistical Analysis

Differences in participant characteristics between SRT groups were assessed with independent-samples t-tests for continuous variables and χ^2^ tests for categorical variables. To determine if measures of brain functional connectivity or gray matter volume differed by SRT group over time, linear mixed models were conducted to account for multiple measurements, with SRT group and time as independent variables and a SRT group-by-time interaction term. ROI-to-ROI functional connectivity and ROI gray matter volume were the dependent variables, which were run in parallel models. Age at baseline, sex, socioeconomic status, handedness, baseline SRT scores, time between timepoints in years, and total brain volume and the head motion summary variable at both timepoints were included as covariates. History of heart conditions and history of smoking were also included as covariates since they are known to increase the prevalence of hearing impairment [36,37] and the risk for dementia [1]. Similarly, due to the prevalent concurrence of hearing impairment and tinnitus [38] and the uncertain effects tinnitus may exert on the brain (for a recent meta-analysis, see [39]), the presence or previous history of tinnitus at baseline was included as a covariate. All variables were entered as fixed factors. Time and participant were entered as repeated variables with an unstructured covariance matrix. Restricted maximum likelihood was used as the estimation method in these models.

Our primary interest was in the interaction of time and SRT group on either functional connectivity or gray matter volume. Significant interactions were followed up with independent-samples t-tests to examine between-group differences at each timepoint and paired-samples t-tests to examine within-group differences between timepoints. Cohen’s *d* effect sizes are reported for these t-tests. Significance in all analyses was set at *p*<.05, two-tailed. Analyses were performed without correction for multiple comparisons because previous literature reports small to medium effect sizes for associations between gray matter volume and hearing ability [12,40-43] and because we carefully selected ROIs *a priori* based on the available literature reviewed above. Statistical analyses were performed using SPSS (IBM Corp. Released 2020. IBM SPSS Statistics for Windows, Version 27.0. Armonk, NY: IBM Corp.).

## Results

### Participant Characteristics

Group comparisons of demographic characteristics (Table 1) revealed that SRT scores were higher (i.e., poorer) in converters compared to non-converters at both baseline (*t*(383.28)=2.65, *p*=.008, *d*=0.25, degrees of freedom were adjusted from 483 because the assumption of equal variances was violated (*F*(4.73), *p*=0.030)), and at follow-up (*t*(438)=27.37, *p*<.001, *d*=2.70). However, it should be noted that the baseline SRT scores for converters were within the cut-off score for “good” hearing ability and that this difference between groups corresponds to a small effect size. Furthermore, linear mixed models reported below remained significant when baseline SRT score was included as a covariate. At baseline, converters also had a greater proportion of individuals who wore hearing aids compared to non-converters (χ^2^(1)=5.95, *p*=0.015, φ=0.12). No other between-group comparisons were significant. Sensitivity analyses on functional connectivity and gray matter volume were conducted by excluding participants who wore hearing aids, as well as by selecting a subset of non-converters to with the same mean baseline SRT score and sample size as converters. The results in these sensitivity analyses were unchanged from the results reported below.

### Functional Connectivity Between Heschl’s Gyrus and the Anterior Insula

**Figure 1.**
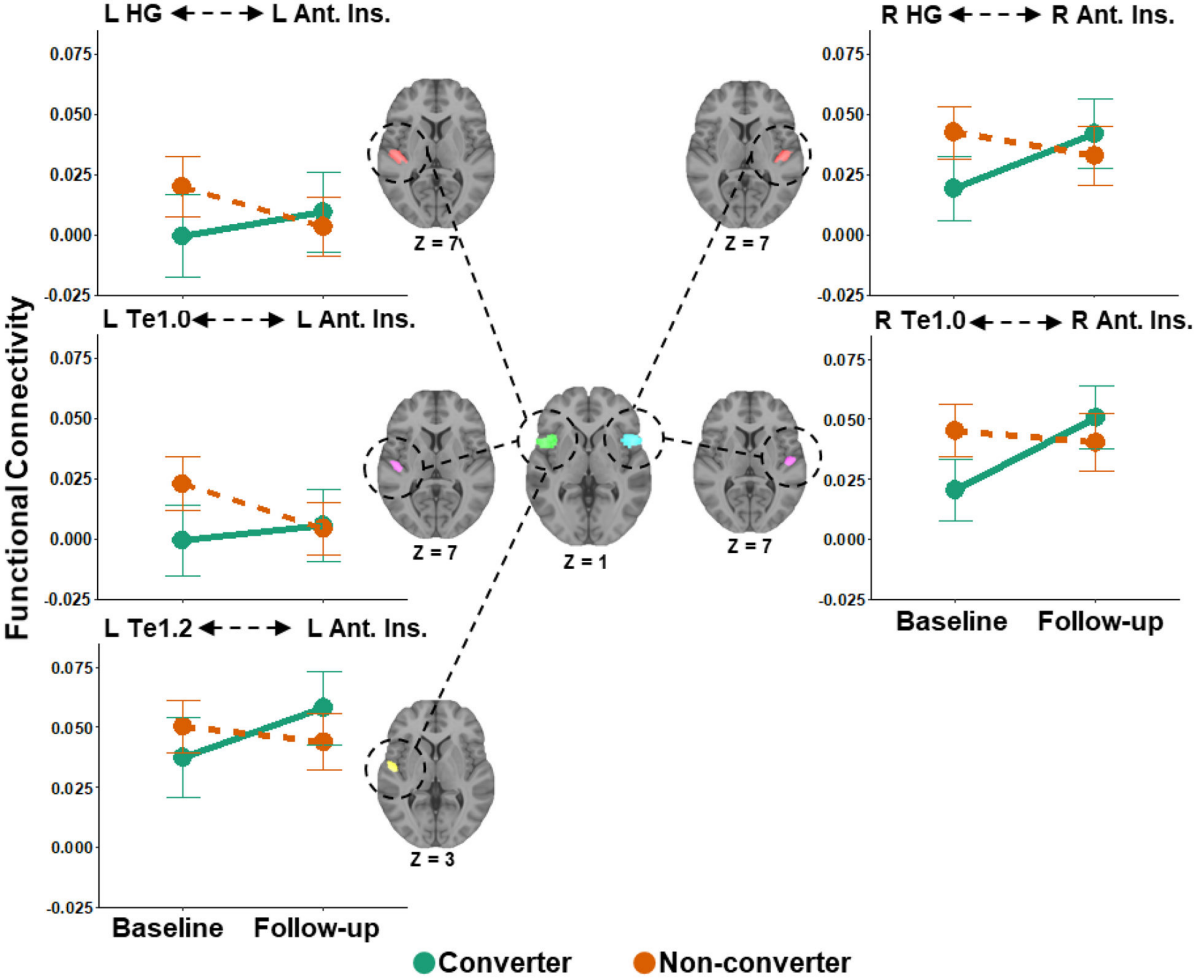
Increased functional connectivity over time between auditory cortex and regions of the cingulo-opercular network in converters, and decreased connectivity over time in non-converters. Functional connectivity was measured between left and right auditory cortex (Heschl’s gyrus and two of its subregions, Te1.0 and Te1.2) and the left and right anterior insula/frontal operculum of the cingulo-opercular network. Converters (green) were those that converted from good to impaired hearing from baseline to follow-up, whereas non-converters (orange) were those that maintained normal hearing. L = Left, R = Right, HG = Heschl’s Gyrus, Ant. Ins. = Anterior Insula. Error bars show 95% confidence intervals.

Linear mixed effect models revealed a significant interaction of SRT group and time on functional connectivity between left Heschl’s gyrus and left anterior insula (Table 3; Figure 1). Independent-samples t-tests show that non-converters had no significant differences in functional connectivity compared to converters at baseline nor follow-up, though the difference at baseline was trending toward significant. Paired-samples t-tests showed that there was a significant decrease in functional connectivity from baseline to follow-up in non-converters and no significant change over time in converters.

A significant interaction of SRT group and time on functional connectivity between right Heschl’s gyrus and right anterior insula was also observed (Table 3; Figure 1). Non-converters had greater functional connectivity than converters at baseline but not at follow-up. There was no significant change in functional connectivity from baseline to follow-up in non-converters, but there was a significant increase in connectivity over time in converters.

### Functional Connectivity Between Heschl’s Gyrus Subregions and the Anterior Insula

Secondary analyses using Heschl’s gyrus subregions as seed ROIs were conducted to determine which subregions may be driving the effects observed in the primary analysis. A significant interaction of SRT group and time on functional connectivity between left Te1.0 and the left anterior insula ROI of the cingulo-opercular network was observed (Table 3; Figure 1). Non-converters had greater functional connectivity than converters at baseline, but connectivity was not different between groups at follow-up. There was a significant decrease in functional connectivity from baseline to follow-up in non-converters, but no significant change in converters.

A significant interaction of SRT group and time was also observed on functional connectivity between left Te1.2 and the left anterior insula ROI (Table 3; Figure 1). There were no significant group differences in functional connectivity at baseline or follow-up. There were also no differences in functional connectivity from baseline to follow-up in non-converters, however, functional connectivity significantly increased in converters from baseline to follow-up.

Lastly, there was a significant SRT group and time interaction on functional connectivity between right Te1.0 and the right anterior insula ROI (Table 3; Figure 1). Non-converters had greater functional connectivity than converters at baseline but not at follow-up. There were no differences in functional connectivity from baseline to follow-up in non-converters, but connectivity increased from baseline to follow-up in converters.

### Functional Connectivity Between Heschl’s Gyrus Subregions and the Hippocampus

**Figure 2.**
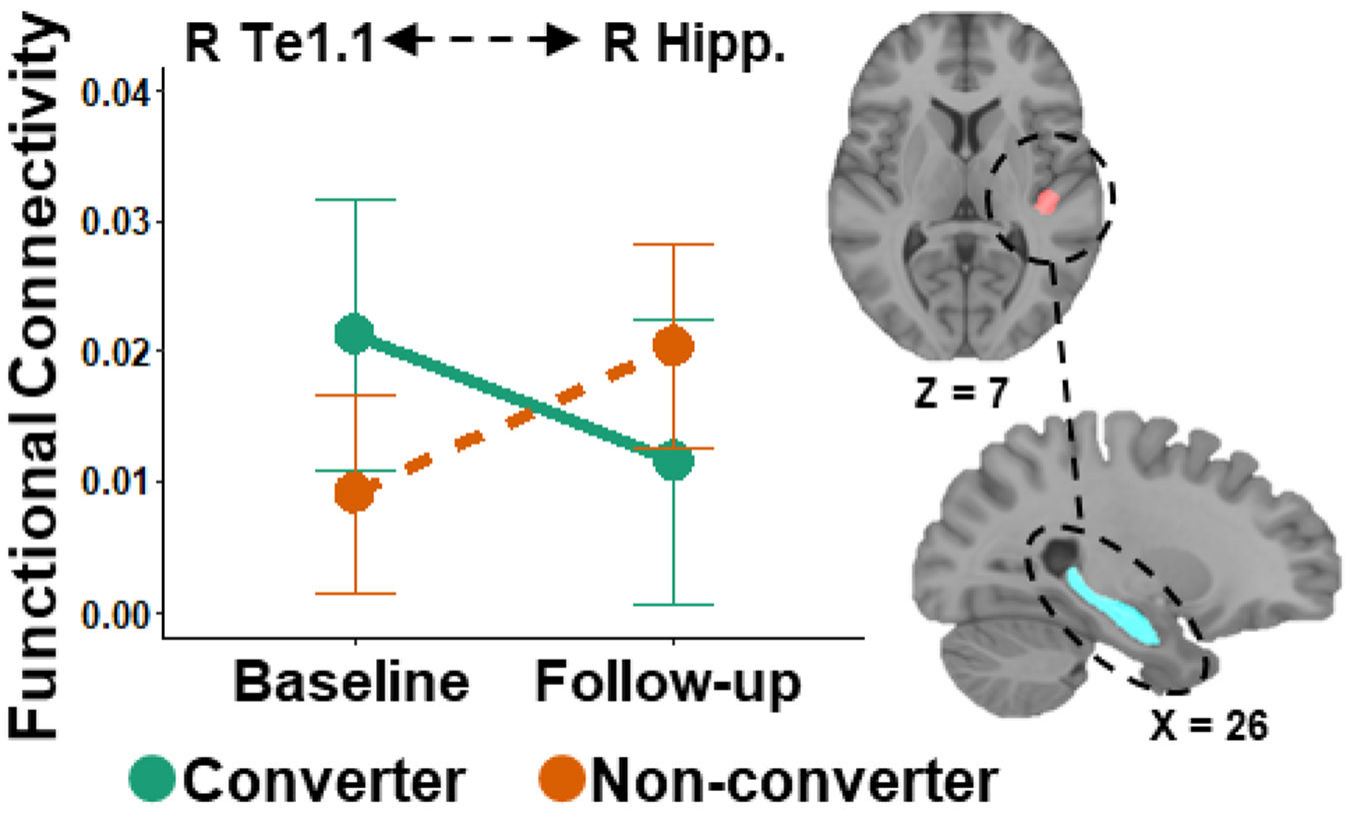
Decreased functional connectivity over time between auditory cortex and the hippocampus in converters. Functional connectivity was measured between right auditory cortex (the Te1.1 subregion of Heschl’s gyrus) and the right hippocampus. Converters (green) were those that converted from good to impaired hearing from baseline to follow-up, whereas non-converters (orange) were those that maintained normal hearing. R = Right, Hipp. = Hippocampus. Error bars show 95% confidence intervals.

There was a significant interaction of SRT group and time on functional connectivity between right Te1.1 and right hippocampus (Table 3; Figure 2). There were no significant differences in functional connectivity between groups at baseline, though this did trend toward greater connectivity in the converter group, and no group differences at follow-up. There was a significant increase in functional connectivity from baseline to follow-up in non-converters, but no significant change in functional connectivity between baseline and follow-up in the converters.

### Gray Matter Volume

**Figure 3.**
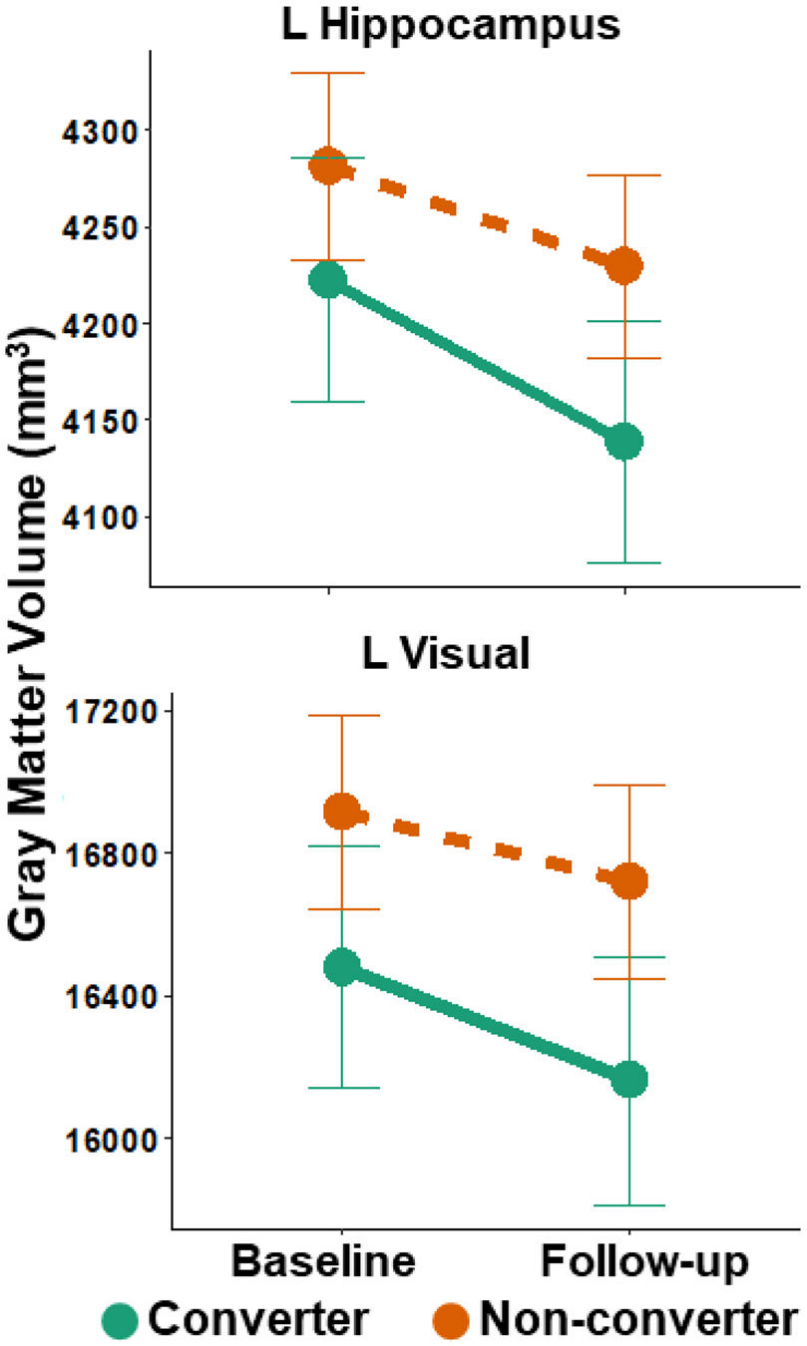
Gray matter volume in the left hippocampus and left lateral superior occipital region showed a significant group-by-time interaction, in which converters had lower gray matter volume at follow-up compared to non-converters, relative to baseline. L = Left. Error bars show 95% confidence intervals.

A significant interaction of SRT group and time on gray matter volume was found in the left hippocampus ROI (Table 4; Figure 3). There were no significant group differences in volume at baseline but greater volume in non-converters compared to converters at follow-up. There were also significant volume decreases in both non-converters and converters over time.

A significant interaction was observed within the left lateral superior occipital ROI (Table 4; Figure 3). There were no significant differences in volume between groups at baseline, though this finding was trending toward significant, but at follow-up, non-converters had significantly greater gray matter volume than converters. Finally, there were significant decreases in gray matter volume between baseline and follow-up in both the non-converters and converters.

## Discussion

This study, to the best of our knowledge, is the first to report longitudinal changes in resting-state functional connectivity within adults who develop impaired hearing. Using a large, community-based sample of participants from the UK Biobank, we show in our primary analysis that the development of a hearing impairment over a two-year period was associated with increased functional connectivity between Heschl’s gyrus and the anterior insula bilaterally. Secondary, exploratory analyses probing functional connectivity from cytoarchitecture-defined subregions within Heschl’s gyrus (i.e., Te1.0, Te1.1, and Te1.2) mirrored the findings from the primary analysis. However, secondary analyses also revealed that functional connectivity from right Te1.1 to the right hippocampus significantly increased from baseline to follow-up in non-converters, while there was no change in functional connectivity over time in converters. In converters, gray matter volume declined within left hippocampus and left visual cortex to a greater degree compared to non-converters. Together, these results show that the transition to a hearing impairment in older adults is accompanied by alterations in brain connectivity and decreases in brain volume within regions associated with attention, memory, and visual processing.

A key finding of this study is that functional connectivity between left Heschl’s gyrus and left anterior insula, and between right Heschl’s gyrus and right anterior insula, relatively increased over time in converters. In comparison, functional connectivity in non-converters tended to decrease over the two-year period. When analyzed by Heschl’s gyrus subregions, connectivity increased from baseline and follow-up between subregion left Te1.2 and the left anterior insula, and between right Te1.0 and right anterior insula, in the hearing impairment converter group. The anterior cingulate and bilateral insular cortices form the cingulo-opercular network, an attention-related network associated with error detection and top-down task maintenance [44,45]. This network is involved in listening to speech in noisy backgrounds, which is an experimental paradigm frequently used, in part, to simulate the effects of hearing loss [46-48]. Our findings align with a previous study that found that hearing loss (measured by pure tone audiometry) in older adults was associated with increased functional connectivity between Heschl’s gyrus and the anterior cingulate of the cingulo-opercular network [20].

This relative increase in connectivity associated with the development of a hearing impairment may reflect a compensatory process of increased use of attentional resources associated with the cingulo-opercular network. Support for this interpretation can be found from speech-in-noise, task-based fMRI literature. Regions within the cingulo-opercular network, particularly the insulae, have greater fMRI BOLD activation to acoustically degraded speech compared to clear speech stimuli [49-51]. Critically, increased activation within the cingulo-opercular network is predictive of correct word identification on subsequent task trials, which may reflect the network’s ability to enhance activity in the task-relevant cortex (i.e., auditory cortex), while suppressing distracting, task-irrelevant information [52]. However, insula engagement to degraded speech may depend on the level of hearing loss. Older adults with poor hearing instead exhibit increased activation in the anterior insula to clear speech, and reduced activation to degraded speech, resembling an inverse U-shape of activation that is dependent upon perceived task difficulty [46].

However, it is unclear why converters had lower functional connectivity than non-converters at baseline in this study. It may be that reduced functional connectivity between these regions precedes the onset of impaired hearing. Taken together, it is possible that with the gradual onset of a hearing impairment, older adults must increasingly rely upon selective attention and performance monitoring processes associated with the cingulo-opercular network for accurate speech perception, even in ideal listening environments, resulting in increased functional connectivity between auditory and cingulo-opercular regions.

Alternatively, it is possible that increased functional connectivity to the anterior insulae reflects altered emotional processing. In addition to its role in attention-related processes, the insula has also been shown to support the integration of affective and interoceptive information [53]. Regarding hearing impairment, one study showed increased cerebral blood flow to the insulae in participants with impaired hearing, which the authors posit may be linked to anxiety and depression that can co-occur with hearing impairment [54]. Another study found decreased activation in the amygdala in response to emotionally salient sounds in those with impaired hearing compared to those with normal hearing [55]. Hearing impairment is associated with increased social isolation and depression, which may in turn affect the functional (as well as structural) properties of the insulae (for a review, see [56] ).

We also report that functional connectivity between the Te1.1 subregion in right Heschl’s gyrus and right hippocampus increased from baseline to follow-up in the non-converter group, whereas in the converter group, connectivity appears to decrease over time, though this comparison was not statistically significant. In addition to its role in long term memory [57], the hippocampus is proposed to be involved in working memory processes [58], although this view is still debated. Using an auditory working memory fMRI task, one study reports increased activation in bilateral hippocampi during the encoding, maintenance, and retrieval phases, as well as greater functional connectivity between right Heschl’s gyrus and right hippocampus during the maintenance and retrieval compared to the encoding phases [59]. Therefore, our finding that connectivity between right hemisphere auditory and hippocampal regions is reduced with the development of a hearing impairment may contribute to the association between hearing loss and memory declines, as those with a hearing impairment may be less able to hold auditory information in working memory. However, this relationship was not examined in our study and should be the focus of future studies.

Lastly, in addition to longitudinal change in functional connectivity, structural brain volume loss over time was also observed. Converters had lower gray matter volume in the left hippocampus ROI at follow-up compared to non-converters. As referenced previously, hearing impairment is associated with reduced volume in right hippocampus [13,17]. However other studies do not separate left and right hippocampal volumes and instead report that volume averaged over both hippocampi is associated with hearing impairment [15,19]. It is unclear why this study found contralateral effects of functional connectivity and volume on the hippocampus. It is possible that hearing impairment affects hippocampal volume bilaterally (indeed, the interaction of SRT group and time on right hippocampal volume was trending toward significant (*p*=.067)), whereas the effect on connectivity may be unilateral.

Furthermore, converters also exhibited greater decline in gray matter volume in the left lateral occipital ROI. This finding mirrors other studies which report lower gray matter volume in occipital cortex associated with poorer hearing both cross-sectionally [13] and longitudinally [18]. Together, these findings add to the growing literature examining how hearing impairment may affect gray matter volume.

Our study assessed hearing ability using SRT scores as this was the sole measure of hearing obtained by the UK Biobank study. However, a majority of published studies investigating associations between hearing and brain measures assess hearing ability using pure tone audiometry. Yet, speech recognition tests may be better predictors of cognitive decline or dementia/Alzheimer’s disease risk than measures of pure tone audiometry (for a review, see [60]). Speech recognition tests also serve as a more ecologically meaningful assessment of hearing ability than listening to tones in silence [61]. The cut-off scores used to categorize hearing ability into normal or impaired hearing groups were defined from a previously published study also using UK Biobank data [27], and have been similarly applied in other studies assessing different data cohorts [62,63]. Critically, a change in SRT scores by 2 dB (i.e., the difference in SRT scores between converters and non-converters at follow-up in this study) reflects an approximate 10 dB increase in pure tone hearing level [27,28]. Thus, we believe our use of SRT cut-off scores to define hearing impairment groups is not only clinically meaningful but also may serve as a compelling measure for investigating biological mechanisms that explain the association between dementia and hearing loss. To better establish the clinical relevance of SRT scores to pure tone audiometry, future studies should collect both PTA and SRT scores and compare the effects of hearing impairment defined by both measures on brain function.

As mentioned previously, hearing impairment and tinnitus often co-occur [38], making it difficult to disentangle the distinct effects of hearing impairment from the effects of tinnitus. A recent meta-analysis revealed that tinnitus is associated with increased regional connectivity within right lateral temporal, inferior parietal, and occipital lobes, and left precuneus [39]. Tinnitus has also been associated with increased connectivity between auditory cortex and regions within the limbic network, including the insula and the parahippocampus [64,65]. However, in a direct comparison between the effects of hearing impairment and tinnitus on both brain volume and white matter integrity, hearing impairment, and not tinnitus, exerted the most impact on brain structure [11]. In our study, the presence or history of tinnitus was included as a covariate and was not a significant predictor in any model. We further report no difference in the prevalence of tinnitus between the converter and non-converter groups. Thus, it is unlikely that the results reported in this paper are driven by tinnitus.

Similar to other studies examining associations between hearing loss and brain volume [12,40-43] and functional connectivity [21], which report small effect sizes, our study also found small effect size differences in resting-state functional connectivity. Not only do these effect sizes correspond to the existing hearing loss literature, but also to studies investigating relationships between functional connectivity and other demographic and behavioral factors of interest. For example, correlations of within- or between-network functional connectivity and age [66-68] or cognitive performance [69-71] exhibit small to medium effect sizes (e.g., Pearson correlations from ~ .10 - .40). Therefore, while functional connectivity-behavior relationships appear to be inherently small, they nevertheless provide important contributions to our understanding of brain-behavior relationships [72], or in the case of this study, our understanding of the association between hearing loss and the brain.

In conclusion, our study shows that conversion to a hearing impairment in older adults is associated with alterations in resting-state functional connectivity between primary auditory cortex and regions linked to attention and memory processing. Specifically, we show that conversion to a hearing impairment over two years results in increased connectivity between bilateral Heschl’s gyrus and anterior insula, and decreased connectivity between right Heschl’s gyrus and right hippocampus. Hearing impairment was further associated reduced gray matter volume in the left hippocampus and left lateral occipital region. However, this study only examined a carefully selected subset of possible brain regions affected by hearing impairment (those with ample structural or functional MRI evidence). It is possible that other brain regions or networks are also altered by hearing impairment, though with equivocal evidence, like the motor cortex and the dorsal attention and default mode networks [8,55,65]. A careful examination of how these regions may be impacted by hearing impairment is needed in future work. Additional studies are also needed to determine if these differences in functional connectivity are related to cognitive performance. Together, our findings of altered connectivity between auditory and higher-order cognitive and visual regions provides evidence for a possible mechanism linking hearing loss and increased risk for dementia.

## Figures and Tables

**Table 1.** Participant characteristics (*N*=440)

	Converters (*n*=163)	Non-converters (*n*=277)	*p*-value	Effect size
Age, mean (*SD*), years	63.11 (6.33)	63.31 (5.50)	.723	0.04
Sex (F/M)	86/77	137/140	.503	0.03
Handedness (%, Right)	87	90	.441	0.04
Hearing aids (%, Yes)	6	1	.015*	0.12
Hx of tinnitus (%, Yes)	34	32	.577	.03
Hx of heart conditions (%, Yes)	14	20	.107	0.08
Hx of smoking (%, Yes)	36	32	.458	0.04
Townsend Deprivation Index	−2.32 (2.35)	−2.44 (2.17)	.581	0.06
Baseline SRT, mean (*SD*), dB	−6.74 (.71)	−6.94 (.84)	.008*	0.25
Follow-up SRT, mean (*SD*), dB	−4.76 (.84)	−6.94 (.79)	<.001*	2.70
Time between visits, mean (*SD*), years	2.21 (.11)	2.22 (.13)	.522	0.06
Head motion at baseline, mean (*SD*)	.10 (.04)	.11 (.05)	.300	0.10
Head motion at follow-up, mean (*SD*)	.11 (.05)	.12 (.05)	.843	0.02

Demographics at baseline and hearing levels for the group of participants who converted to a hearing impairment between study visits (converters) and for the group who did not convert (non-converters). Significance of continuous variables (age, Townsend, SRT, time, head motion) were tested using independent samples t-tests; dichotomous variable distributions (sex, handedness, hearing aids, tinnitus, and history of heart conditions or smoking) were tested using Chi-square tests. Cohen’s *d* and Phi effect sizes were computed for continuous and dichotomous variables, respectively. *Indicates significant group differences p < 0.05. Hx = History, SRT = Speech reception threshold.

**Table 2.** ROI names and MNI center of mass coordinates

	X	Y	Z	Size (voxels)
L Heschl’s Gyrus	−45	−20	7	2507
L Te1.1	−39	−29	10	1292
L Te1.0	−48	−18	6	984
L Te1.2	−52	−6	1	1119
R Heschl’s Gyrus	46	−17	7	2223
R Te1.1	41	−25	10	1622
R Te1.0	50	−13	5	1209
R Te1.2	55	−3	−1	883
Cingulo-opercular Network (CON)				
Anterior Cingulate Cortex	0	22	35	1063
L Anterior Insula/Frontal Operculum	−44	13	1	446
R Anterior Insula/Frontal Operculum	47	14	0	388
Visual Network				
Medial	2	−79	12	79224
Occipital	0	−93	−4	48712
L Lateral	−37	−79	10	24832
R Lateral	38	−72	13	33968
L Hippocampus	−25	−23	−14	6127
R Hippocampus	27	−21	−14	5625

L = left, R = right

**Table 3.** Significant SRT group by time interactions on functional connectivity and post-hoc tests

Left Heschl’s Gyrus-Left Anterior Insula (*F*(1,438.25)=4.16, *B*=.03, *p*=.042)	*t*-value	*p*-value	Cohen’s *d*
Non-Converters vs Converters at Baseline	1.89	.060	0.13
Non-Converters vs Converters at Follow-up	−0.61	.542	0.06
Change From Baseline to Follow-up in Non-Converters	2.16	.032*	0.13
Change From Baseline to Follow-up in Converters	−0.99	.325	0.08
Right Heschl’s Gyrus-Right Anterior Insula (*F*(1,438.30)=7.29, *B*=.03, *p*=.007)			
Non-Converters vs Converters at Baseline	2.61	.009*	0.26
Non-Converters vs Converters at Follow-up	−0.91	.361	0.09
Change From Baseline to Follow-up in Non-Converters	1.29	.200	0.08
Change From Baseline to Follow-up in Converters	−2.53	.012*	0.20
Left Te1.0-Left Anterior Insula (*F*(1,438.21)=4.44, *B*=.02, *p*=0.036)			
Non-Converters vs Converters at Baseline	2.50	.013*	0.25
Non-Converters vs Converters at Follow-up	−0.16	.877	0.02
Change From Baseline to Follow-up in Non-Converters	2.66	.008*	0.16
Change From Baseline to Follow-up in Converters	−0.69	.490	0.05
Left Te1.2-Left Anterior Insula (*F*(1,438.07)=4.96, *B*=.03, *p*=0.026)			
Non-Converters vs Converters at Baseline	1.32	.187	0.13
Non-Converters vs Converters at Follow-up	−1.46	.145	0.14
Change From Baseline to Follow-up in Non-Converters	0.94	.347	0.05
Change From Baseline to Follow-up in Converters	−2.05	.042*	0.16
Right Te1.0-Right Anterior Insula (*F*(1,438.28)=8.96, *B*=.04, *p*=.003)			
Non-Converters vs Converters at Baseline	2.78	.006*	0.27
Non-Converters vs Converters at Follow-up	1.09	.276	0.11
Change From Baseline to Follow-up in Non-Converters	0.67	.502	0.04
Change From Baseline to Follow-up in Converters	−3.44	<.001*	0.27
Right Te1.1-Right Hippocampus (*F*(1,437.96)=4.92, *B*=− .02, *p*=0.027)			
Non-Converters vs Converters at Baseline	−1.87	.062	0.19
Non-Converters vs Converters at Follow-up	1.32	.188	0.13
Change From Baseline to Follow-up in Non-Converters	−2.08	.039*	0.13
Change From Baseline to Follow-up in Converters	1.21	.230	0.09

Non-converters vs converters comparisons at each timepoint were conducted with independent-samples *t*-tests and change from baseline to follow-up comparisons in each group were conducted with paired-samples *t*-tests. *p<.05.

**Table 4.** Significant SRT group by time interactions on gray matter volume and post-hoc tests

Left Hippocampus ROI (F(1,430.86)=6.28, B=−30.29 p=.013)	*t*-value	*p*-value	Cohen’s *d*
Non-Converters vs Converters at Baseline	1.46	.146	0.14
Non-Converters vs Converters at Follow-up	2.26	.024*	0.22
Change From Baseline to Follow-up in Non-Converters	7.45	<.001*	0.45
Change From Baseline to Follow-up in Converters	8.47	<.001*	0.66
Left Lateral Superior Occipital ROI (*F*(1,437.12)=5.02, *B*=−114.74 *p*=.026)			
Non-Converters vs Converters at Baseline	1.94	.053	0.19
Non-Converters vs Converters at Follow-up	2.47	.014*	0.24
Change From Baseline to Follow-up in Non-Converters	5.90	<.001*	0.35
Change From Baseline to Follow-up in Converters	7.65	<.001*	0.60

Non-converters vs converters comparisons at each timepoint were conducted with independent-samples *t*-tests and change from baseline to follow-up comparisons in each group were conducted with paired-samples *t*-tests. *p<.05.
